# Detection of *Sutterella* spp. in Broiler Liver and Breast

**DOI:** 10.3389/fvets.2022.859902

**Published:** 2022-03-30

**Authors:** Sophia Derqaoui, Mohammed Oukessou, Kawtar Attrassi, Fatima Zahra Elftouhy, Saadia Nassik

**Affiliations:** ^1^Unit of Avian Pathology, Department of Veterinary Pathology and Public Health, Agronomy and Veterinary Medicine Institute Hassan II, Rabat, Morocco; ^2^Unit of Physiology and Therapeutics, Department of Veterinary Biological and Pharmaceutical Sciences, Agronomy and Veterinary Medicine Institute Hassan II, Rabat, Morocco

**Keywords:** *sutterella* spp., poultry foodstuffs, broiler, food safety, human contamination

## Abstract

*Sutterella* sp. is a gram-negative, microaerophilic bacterium that is particularly resistant to bile acids. It has recently been associated with several human pathologies such as inflammatory bowel disease, asthma, diabetes, and autism. Indeed, susceptibility patterns to ciprofloxacin and erythromycin, combined with resistance to metronidazole, indicate that *Sutterella wadsworthensis* patterns are closer to those of *Campylobacter*. The objective of this study is to identify, for the first time, *Sutterella* spp. in the liver and breast of broiler chickens by quantitative real-time PCR (qPCR). Liver, breast, and cecal content samples were taken from 25 birds and frozen at −20°C until analyzed. The main results showed that *Sutterella* sp. is part of the cecal microbiota of 48% of the birds and present in the liver and breast of, respectively 20 and 40% of the chicks with a variable Cq. We, therefore, conclude that *Sutterella* sp. exists in poultry and poultry meat and that foodstuffs of poultry origin might be considered as a potential source of contamination for humans.

## Introduction

Nuanced in the beginning with *Campylobacter* in 1996, *Sutterella* sp. is a gram-negative, non-spore-forming, asaccharolytic, microaerophilic bacterium, particularly resistant to bile acids (1). In 1997, this bacterium was isolated from patients with appendicitis, peritonitis, or rectal or perirectal abscesses (2). Isolated by the filter method from the feces of patients with gastrointestinal disorders, *Sutterella* sp. was included in the *Campylobacter* taxon along with *Campylobacter jejuni* and *Campylobacter coli* (3). Subsequently, it was identified as a putative human pathogen and phylogenetically differentiated from *Campylobacter* mainly by its bile resistance, cell wall fatty acid profile, and 16S rRNA sequencing, but it is still more likely to be involved in serious infections than *C. gracilis* (4, 5). However, mystery still surrounds *Sutterella* sp. as its average abundance in the duodenal mucosa can reach 19% in children with celiac disease and healthy children (6) and it has a substantial relative abundance in the duodenum of adults with a decreasing gradient toward the colon. This microaerophilic bacterium has recently been associated with several human pathologies (7). Indeed, compared to healthy individuals, an overabundance of *Sutterella* spp. can cause allergic disease (8) due to excessive production of bacterial toxins (9). On ileal and cecal biopsies, this little-known bacterium comes to the fore as a major component of the mucoepithelial microbiota (7%) in 52% of children with both autism and gastrointestinal dysfunction (AUT-GI), but *Sutterella* sp. is absent in children with gastrointestinal disorders only (10). In Rett syndrome, the change in the microbiota composition in favor of a few germs including *Sutterella* spp. may also be involved (11). Recently, *Sutterella* sp. has been linked to obesity in adults (12), children, and adolescents (13). The few investigations, to our knowledge, that followed have identified 10 species of the genus *Sutterella* including *S. wadsworthensis, S. parvirubra, S. morbirenis*, and *S. stercoricanis* isolated from the human gastrointestinal tract, canine fecal matter (4, 5, 14), and cattle (15) and poultry intestinal content (16).

According to the Food and Agriculture Organization of the United Nations (FAO), poultry contributes to 39% of the world production of animal proteins with 132 million tons of white meat in 2019 (17). Thereafter, the per capita demand for poultry meat is expected to increase by 271% in South Asia, 116% in Eastern Europe and Central Asia, 97% in the Middle East and North Africa, and 91% in East Asia and the Pacific between 2000 and 2030 (18). Consequently, this increase may lead to the degradation of the hygienic and microbiological qualities of white meat. Thus, poultry and poultry meat will constitute a potential source of contamination for humans (19) during the entire production process, mainly at the slaughterhouse level, via germ transmission from the intestinal content to the carcass (20, 21). As previously mentioned in the European Union Report, the post-slaughter prevalence of *Salmonella* in neck skin and thigh is 16% and 10%, respectively, with 53.8% serotypes in favor of *Salmonella* Infantis (22), one of the strains responsible for human salmonellosis (23) and is controlled since 2003 at breeder hens as well as at broilers (24), but who represents the highest multidrug resistance scores in 2010 in the USA (25). Moreover, during all stages of the slaughter process, 50 and 42% of the carcasses are *Campylobacter* positive with <3.0 log CFU/g and between 3.0 and 4.0 log CFU/g, respectively (26). Therefore, the presence of *Sutterella* spp. in the poultry gut could tip toward human contamination as a result of the non-conformance to the Hazard Analysis and Critical Control Point (HACCP) procedure at slaughter or the translocation of bacteria through the intestinal barrier to the systemic environment. Although the pathogenicity of *Sutterella* spp. is not well-elucidated to date, plasma IgG antibodies to *Sutterella wadsworthensis* proteins were detected in 34.78% of *Sutterella* sp. infections. This suggests that *Sutterella* sp. is hypothetically pathogenic in humans (10).

The objective of this study is to demonstrate the presence of *Sutterella* spp. in the broiler chicken's intestinal contents, liver, and breast by quantitative real-time PCR (qPCR) for the first time.

## Materials and Methods

### Sampling Design and Sample Collection

Samples were taken from five live poultry markets (A to E) supplied by different farmers and where live poultry are assembled and held for sale and slaughter. Five 42-day-post-hatch (d-p-h) live chicks of Ross 308 strain, weighing 1,950 g in average, were randomly taken from each market and transferred to our premises. All chicks' feed was formulated to meet nutrient requirements according to the Ross 308 manual with a growth promoter antibiotic (avilamycin, enramycin, or flavomycin) or alternative. Birds were humanely sacrificed by dislocation of the cervical vertebrae and dissected individually. From each cadaver, cecal content, the right liver lobe, and a breast sample were collected in a sterile manner to avoid cross-contamination of the samples. The samples were immediately stored at −20°C till analyzed.

### DNA Extraction

Extraction of bacterial DNA from the collected samples was performed using a commercial column-based kit Invitrogen™ PureLink™ Microbiome DNA Purification Kit (Life Technologies, Thermo Fisher Scientific, Foster City, CA, USA) as described in the manufacturer's user guide. Briefly, the cecal contents were thawed and transposed into swabs while 25 mg of the livers and brevis was directly put into individual tubes to initiate lysis.

The cecal swabs were placed into sterile microtubes and vortexed after adding 500 μl of phosphate-buffered saline (PBS) solution. Thereafter, 200 μl of this solution was added to 20 μl of proteinase K along with 200 μl of lysis solution and subsequently incubated in a water bath at 55°C for 10 min. After incubation and centrifugation, 200 μl of 100% ethanol was added to the sample, and the final solution was vortexed. For the liver and breast, 180 μl of PureLink® genomic digestion buffer and 20 μl of proteinase K were added to the tube with the 25 mg of collected tissue and incubated at 55°C in a water bath with occasional vortexing until lysis was complete (1 to 4 h). Then the lysate is centrifuged at 15,000 rpm for 3 min at room temperature, and the supernatant is transferred to a new sterile tube. After adding 20 μl of ribonuclease A (RNase A) to the lysate, the obtained solution is incubated at room temperature for 2 min. Two hundred microliters of PureLink® Lysis/Genomic Linkage Buffer is added and vortexed before adding 200 μl of 97% ethanol. The 620 μl of obtained lysate solution prepared for both swabs and tissues is then placed in collection tubes and centrifuged at 10,000 rpm for 1 min at room temperature. The collection tubes are then replaced, and two rounds of washing are subsequently performed using the washing solutions provided with the kit. The DNA is finally recovered after the elution step and stored at −20°C until use.

### qPCR Test

Quantification of *Sutterella* spp. from samples of intestinal contents, livers, and breasts was performed using previously described methods (27–29). qPCR was performed in duplicate reactions so that each well of the plate contained 10 μl of the reaction at a rate of 5 μl of the test sample and 5 μl of the one-step mix. The prepared mix includes nuclease-free water, forward and reverse primers for each gene (Table 1), and SYBR Green (Applied Biosystems, Life Technologies, Burlington, ON, Canada). Once a plate is sealed with an adhesive, it is placed in a thermal cycler (Agilent AriaMx Real-Time PCR System) according to *Sutterella* sp. primer melt temperature (Table 2). The values obtained are expressed as quantification of qPCR cycle numbers (Cq). In the absence of any previously defined ‘Cq cut-off' as for *Salmonella* and *Campylobacter*, for example, any sample with a Cq above 35 is considered as negative.

**Table 1:** Primer sequences of *Sutterella* spp. (10).

**Gene**	**Primers**	**GenBank** **accession No**.
*Sutterella* spp.	F: CGCGAAAAACCTTACCTAGCC	GCA_905204555.1
	R: GACGTGTGAGGCCCTAGCC	

**Table 2:** Cycling mode of *Sutterella* spp. according to primer melt temperature (30).

**Step**	**Incubation**		**Cycles**
	**Temperature**	**Time**	
UDG activation	50°C	2 min	Hold
Dual-lock DNA polymerase	95°C	2 min	Hold
Denature	95°C	15 s	40
Anneal	50–60°C	15 s	
Extend	72°C	1 min	

## Results

The main results of our study show that *Sutterella* sp. was detected in the cecal contents of 12 chicks (48%) with a Cq ranging from 17.43 to 31.39. In the liver, 5 of 25 autopsied birds were positive for *Sutterella* spp. with Cq varying from 13.97 to 33.41. For breast samples, 10 of the 25 chickens autopsied were positive for *Sutterella* spp. with Cq values between 22.62 and 34.75 (Table 3). It should be noted that the obtained Cq values are inversely proportional to the bacterial load of the tested samples. That is, when the Cq is high, the sample's load in bacterial DNA is low, and when the Cq is low, the bacteria are amply present in the sample.

**Table 3:** Presence of *Sutterella* spp. with corresponding Cq values in broiler cecal content, liver, and breast.

**Live poultry market**	**Samples**	**Cecal content**	**Liver**	**Breast**
A	1	ND	ND	ND
	2	ND	ND	ND
	3	ND	ND	ND
	4	ND	ND	ND
	5	ND	ND	ND
B	6	*21.63*	ND	ND
	7	*18.92*	ND	ND
	8	*18.35*	ND	ND
	9	*21.13*	ND	*28.85*
	10	ND	*33.26*	*34.40*
C	11	*31.39*	ND	*27.24*
	12	ND	ND	*32.83*
	13	ND	ND	ND
	14	*17.43*	ND	ND
	15	ND	ND	ND
D	16	ND	ND	ND
	17	ND	ND	ND
	18	ND	ND	ND
	19	*28.72*	*13.97*	*22.62*
	20	ND	ND	*34.75*
E	21	*23.59*	33.41	ND
	22	*19.69*	*31.04*	*30.62*
	23	*26.62*	ND	*30.79*
	24	*19.64*	*29.82*	*30.42*
	25	*19.07*	ND	*30.77*
**Contaminated/Total**	12/25	5/25	10/25

*ND: not detected.

## Discussion

Recent advances in DNA sequencing have made the comparison of the composition of the gut microbiota, in the context of human diseases, possible. Indeed, it has been suggested that changes in the composition of the gut microflora might be associated with alterations in the severity of various human autoimmune diseases such as inflammatory bowel disease (31), asthma (32), allergies (33), diabetes (34), rheumatoid arthritis (35), and systemic lupus erythematosus (36). As a result, the gut–brain link is currently being explored experimentally in relation to various disorders including those of the central nervous system in humans (37) such as autism (10, 37), amyotrophic lateral sclerosis (38), and Rett syndrome (11) and those related to the gastrointestinal tract (GIT) (7). Several bacteria are indirectly incriminated in the occurrence of this array of alterations including *Sutterella* spp. Indeed, out of 655 tested individuals, 18.47% with metabolic syndrome (MS) were *Sutterella* spp. positive while healthy ones were *Sutterella* spp. negative (39). This suggests that the abundance of this bacterium in the gut of patients with MS and its absence in healthy individuals could be due to a possible contamination or as a consequence to an imbalance in the gut microbial community. For example, a dysbiosis caused, in part, by increasing *Sutterella* sp. abundance in the middle-aged and elderly patients' GIT with cardiovascular disease can be related to smoking (40). In this respect, one of the most common forms of contaminations, when it comes to bacteria and GIT, is the ingestion of infected foodstuffs of animal origin such as poultry meat. As example, foodborne illnesses caused by *Salmonella* spp. or *Campylobacter* spp. are widely common due to contaminations of edible parts of poultry as a result of poor slaughter hygiene (19, 41) or systemic dissemination (42). In China, with a prevalence of 15.3%, *Salmonella* is considered to be the highest foodborne pathogen transmitted by raw meat including white meat (43).

Our study allowed us to show, for the first time, the presence of *Sutterella* spp. in chicken's edible parts, namely, liver and breast. The results of this first detection suggest that when the bacterium is present in the cecal content, a passage to the liver and the breast by bacterial translocation would seem possible. Passage of *Sutterella* spp. across the intestinal barrier would challenge intestinal integrity and permeability in birds. However, there is risk that a poultry carcass could be contaminated as a result of poor slaughter hygiene, except for our study since the conditions of sacrifice and sampling were heavily under control. The results of this study lead to three hypotheses:

1. The presence of *Sutterella* spp. in, only, the intestinal contents of few birds suggests that their intestinal junctions are tightly packed, preventing its translocation outside the GIT. Indeed, sequencing of the poultry gut microbiota highlights the presence of this bacterium in few chicks (16).

2. The identification of *Sutterella* spp. in cecal contents, liver, and breast at varying bacterial loads from the most to the least loaded organs, respectively, could be due to the passage of the bacteria via the intestinal barrier to the liver and then to the muscle. This possible systemic dissemination refers to that of *Salmonella* spp. as reported earlier (43).

3. The detection of *Sutterella* spp. in the liver only and/or in the breast suggests that this bacterium may have an intermittent excretion, hence its very low bacterial load (Cq: 31.39) or its absence from the cecum.

On the other hand, the high loads of this bacterium in chicken cecal content could be associated to an eventual microbiota dysbiosis which allows *Sutterella* spp.'s proliferation in broiler gut or could be related to feed supplementation (antibiotics or natural alternatives) since sacrificed chickens were from different farms. In that, 5 or 10% inclusion level of *Tenebrio molitor* (TM) in poultry meal diets revealed the presence of *Sutterella* spp. in cecal content compared to 15% TM supplementation and to the control group where this bacterium was absent (16).

Although *Sutterella* sp. has been differentiated from *Campylobacter gracilis* (4, 5), susceptibility patterns to ciprofloxacin and erythromycin, combined with resistance to metronidazole, indicate that *Sutterella wadsworthensis* patterns are closer to those of *Campylobacter* rather than to those of obligate anaerobes (44). In addition, *Sutterella* spp. is among the resistant bacteria implicated in regressive autism (45) potentially as a result of excessive production of bacterial toxins (46).

In a recent report, therapeutic remission of ulcerative colitis (UC) patients was linked to IgA degradation by *Sutterella* spp. proteases, which seem to mediate the effects of this bacterium on human health (14). Thus, rather than directly inducing inflammation, *Sutterella* spp. may alter the functionality of the intestinal antibacterial immune response, particularly in its ability to limit intracellular bacterial species (47). In mice, the presence of *Sutterella* spp. was strictly related to the group with depressive-like behaviors, suggesting that it may be associated with the development of depression (48). Recently, *Sutterella wadsworthensis* was incriminated as the cause of bacteremia in three immunocompetent patients who underwent intra-abdominal surgery (44) along with *C. gracilis* responsible for fatal bacteremia (49), which suggested the pathogenic potential of these bacteria.

Although there have been few studies identifying *Sutterella* spp. as a commensal bacterium in broilers, its role and pathogenicity remain unknown. The presence of this bacterium in the intestinal tract of humans may result from the ingestion of contaminated food, particularly poultry meat, and dysbiosis might underlie its pathogenicity.

## Conclusion

This first-time detection of *Sutterella* spp. in the liver and breast of broiler chickens opens a new area for further investigations on the pathogenicity of this bacterium and its relationship with human and animal health. It would be of high interest to proceed to 16S rRNA sequencing of the *Sutterella* spp. positive samples in order to (1) determine whether this strain is specific to poultry gut microbiota or common to other species and (2) determine its mode of transmission and minimal acceptable safe concentration in foodstuffs.

## Data Availability Statement

The datasets presented in this study can be found in online repositories. The names of the repository/repositories and accession number(s) can be found in the article/supplementary material.

## Ethics Statement

The animal study was reviewed and approved by Pr El ALLALI Pr RHALEM Pr ELBERBRI Pr ELYAKINE Pr SRAIRI Pr KICHOU Pr BOUSLIKHANE.

## Author Contributions

SD and FE collected samples used in the study after an autopsy. SD, FE, and KA performed the qPCR technique. SD and SN analyzed the qPCR data. SD wrote the original draft. SN and MO revised and edited the draft and generated the final version of the manuscript. All authors contributed to the article and approved the submitted version.

## Conflict of Interest

The authors declare that the research was conducted in the absence of any commercial or financial relationships that could be construed as a potential conflict of interest.

## Publisher's Note

All claims expressed in this article are solely those of the authors and do not necessarily represent those of their affiliated organizations, or those of the publisher, the editors and the reviewers. Any product that may be evaluated in this article, or claim that may be made by its manufacturer, is not guaranteed or endorsed by the publisher.
